# Influencing factors of coking coal dust explosion pressure and flame and effect of inert dust on its explosion suppression

**DOI:** 10.1038/s41598-022-21859-0

**Published:** 2022-10-20

**Authors:** Tianqi Liu, Xuan Zhao, Weiye Tian, Ruiheng Jia, Ning Wang, Zhixin Cai

**Affiliations:** School of Safety Engineering, Shenyang Aerospace University, Shenyang, 110136 Liaoning People’s Republic of China

**Keywords:** Chemistry, Energy science and technology, Engineering, Chemical engineering

## Abstract

Coking coal is a precious resource in the world and an important raw material for the production of steel, but it is easy to cause explosion accidents in the process of coking coal mining, which is very detrimental to safe production. In order to reveal the influencing factors of coking coal dust explosion intensity and the suppression effect of inert dust on coking coal dust explosion, an experimental study was carried out in this paper. The results show that the particle size and the mass concentration of coal dust have a great influence on the explosion pressure and flame. By analyzing the suppression effects of NaCl, KCl, and NH_4_H_2_PO_4_ on coking coal dust explosion, it is got that NH_4_H_2_PO_4_ has the best explosion suppression effect. When the mass percentage of NH_4_H_2_PO_4_ mixed into coking coal dust increases to 60%, the maximum explosion pressure decreases by 0.47 MPa, and the maximum flame length decreases by 0.50 m. As the particle size of NH_4_H_2_PO_4_ decreases, the explosion intensity continue to decrease. When the particle size of NH_4_H_2_PO_4_ is 0 ~ 25 μm, and the mass percentage of NH_4_H_2_PO_4_ mixed into coking coal dust is 50%, the explosion doesn’t occur anymore.

## Introduction

Coking coal is a valuable coal resource that is widely distributed in the United States, Russia, China, Japan and many other countries. Coking coal can be used to produce steel, so it plays a very important role in developing the economy. In China, the distribution of coking coal is uneven, mainly in North China and East China. Among them, Shanxi Province has the largest reserves of coking coal resources. Relevant coal mining and production enterprises attach great importance to the problem of coal dust explosion, because in the event of a coal dust explosion accident, the ventilation system of the mine will be destroyed, causing the company to stop production and bring huge economic losses^1^. At present, the research focus of coal dust explosion mainly focuses on explosion pressure and flame^2,3^. Studies have shown that in the coal mine tunnels, due to the presence of deposited coal dust, it is easy to cause secondary explosions. In addition, because there are many obstacles in the tunnels, the explosion intensity will be greatly increased under the action of the obstacles^4,5^. In order to avoid the occurrence of coal dust explosion accidents, the use of inert dust to suppress explosions has become a very effective method^6,7^.

In the process of coal dust explosion, combustible volatile gas will be released. Therefore, coal dust explosion not only includes particle phase explosion, but also gas phase explosion^8–15^. However, there is also a big difference between a combustible gas explosion and a coal dust explosion. The main difference between gas explosion and coal dust explosion is that coal dust explosion is more intense, mainly because the collision between particles will release more energy and cause more serious damage^16–18^. In addition, there are many influencing factors of coal dust explosion, which is one of the main reasons why scholars cannot accurately control the explosion. Eckhoff^19^ researched on the influence of the physical properties and chemical properties of dust on the explosion intensity. Houim^20^ compared the difference between gas explosion and dust explosion, and obtained the influencing factors of the explosion flame. Kosinski^21^ obtained the formation process of dust explosion by using simulation method, the accuracy of the simulation result was well verified. The research results of the above scholars have played a certain role in grasping the influencing factors of coal dust explosion, but it is not enough for a comprehensive understanding of the change law of coal dust explosion.

In addition, in order to control the occurrence of coal dust explosions and reduce the hazards of explosions, many methods have been proposed, including explosion suppression, explosion venting and explosion isolation. The use of inert dust to suppress coal dust explosion is one of the earliest proposed methods to control coal dust explosion. By spreading inert dust in the tunnels, it is found that the occurrence of secondary explosion can be effectively suppressed, and the risk of depositing coal dust participating in secondary explosion can be greatly reduced^22–24^. The study also found that different types of inert dust have different explosion suppression effects, but the cost of inert dust also needs to be considered when selecting an efficient inert dust to suppress explosions. Even for the same type of inert dust, if its particle size, degree of dispersion, humidity, and spreading range are different, the effect of suppressing explosions is also different^25–29^. Inert dust with better explosion suppression effect usually not only does not participate in the explosion, but also absorb heat during the suppression process, and can release some gases and solid products that help to suppress the explosion, so as to achieve better explosion suppression effect^30,31^. In order to obtain a better explosion suppression effect, the research on inert dust is very important, and at present, there is no effective explosion suppression method that can completely suppress the explosion of coking coal dust at home and abroad. Therefore, this paper will conduct experimental research on the suppression of coking coal dust explosion, trying to provide more support for the research on explosion suppression.

In previous studies, the authors found that coal dust explosion pressure and flame have many influencing factors, indicating that coal dust explosion intensity is uncontrollable. The authors also found that coal dust with different degrees of metamorphism has different explosion intensities, and the particle size, coal dust cloud mass concentration, and ignition delay time have a great influence on the explosion intensity^32–34^. At the same time, there is a great correlation between the ignition characteristics of coal dust cloud and the characteristics of explosion intensity^35,36^. Therefore, in this paper, in order to further explore the suppression effect of inert dust on coal dust explosion, coking coal was selected as coal dust sample, NaCl, KCl and NH_4_H_2_PO_4_ were selected as inert dust samples, explosion pressure and flame were used to characterize explosion intensity, and influencing factors of explosion pressure and flame were studied. At the same time, the suppression effect of inert dust on explosion intensity was analyzed. The results of this paper are of great significance for understanding the influencing factors of coking coal explosion intensity and the explosion suppression effect of inert dust.

## Experimental apparatus and samples

### Experimental apparatus

**Figure 1:**
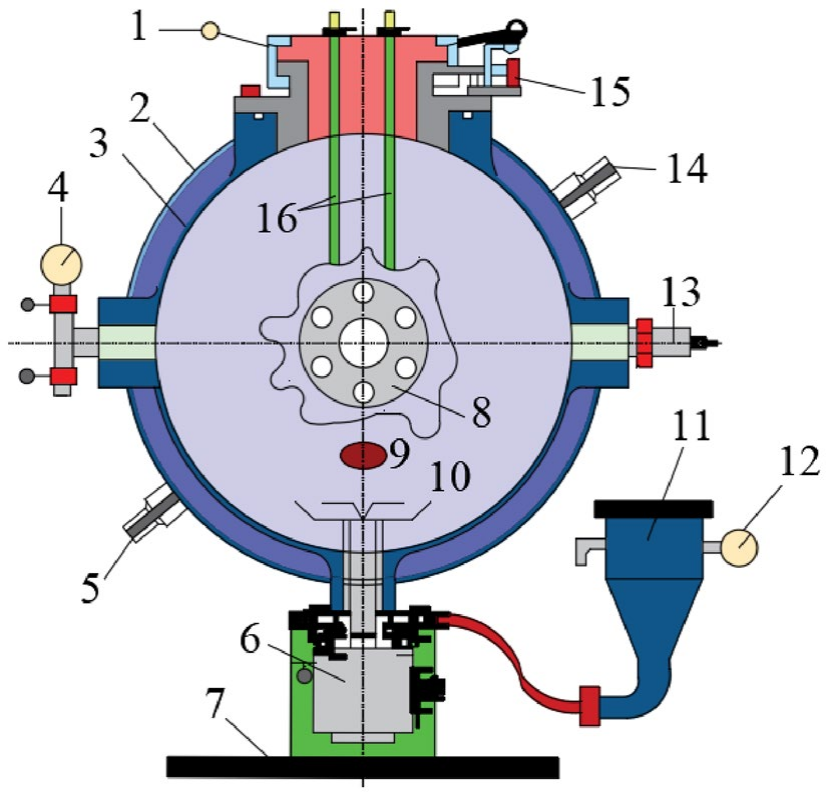
Structure of experimental apparatus drawn with CAD software 2018 version. (URL link: https://www.autodesk.com.cn/) 1 sealing cap; 2 outer side of mezzanine; 3 inside of mezzanine; 4 vacuum gauge; 5 outlet of circulating water; 6 mechanical two-way valve; 7 base; 8 observation window; 9 vacuum hole; 10 dispersion valve; 11 dust storage tank; 12 pressure gauge; 13 pressure sensor; 14 inlet of circulating water; 15 safety limit switch; 16 ignition rod.

In this paper, the explosion pressure and flame of coal dust are used to characterize the explosion intensity, so the experimental apparatus used include explosion pressure apparatus and explosion flame apparatus. As shown in Fig. 1, it shows the coal dust explosion pressure test apparatus. It can be used to obtain the curve of the pressure change during the explosion process. By analyzing the data on the curve, the maximum value of the explosion pressure can be obtained. The maximum value of the coal dust explosion pressure can be abbreviated as *P*_max_. Because the internal volume of this explosion pressure apparatus is 20 L and the interior is a spherical structure, it is also called a 20 L spherical coal dust explosion apparatus. In general, the main advantages of this spherical experimental apparatus are summarized as follows.

- (1) Firstly, it can spray dust automatically by using computer control system, and the explosion experiment process can be remotely controlled to ensure the safety of personnel.
- (2) Secondly, the explosion pressure data can be automatically collected and aggregated, which is convenient for the processing and analysis of experimental data.
- (3) Thirdly, automatic water circulation system can be used to cool down the interior of the apparatus quickly after the explosion, which can save time for the next coal dust explosion experiment.

In addition, in this paper, authors still use the coal dust explosion flame test apparatus, the structure and test method of this apparatus have been introduced in detail in the literature^36^, and will not be introduced here. By conducting the coal dust explosion flame test, the propagation distance of the coal dust explosion flame along the direction of the pipe body can be obtained, the flame length at different times can be recorded, and finally the maximum propagation length of the coal dust explosion flame can be obtained, which is usually abbreviated as is *l*_max_. Therefore, *P*_max_ and *l*_max_ can be used to quantitatively describe the explosion intensity of coal dust, and they can also be used to study the influencing factors of explosion pressure and flame and the suppression effect of inert dust on explosion intensity.

### Experimental samples

#### Coking coal dust sample

The coking coal sample selected in this paper comes from the Lu’an Coal Mine in Shanxi Province. As shown in Table 1, it presents data on the main components of the coal sample. According to the proximate analysis result of the coal sample, it can be found that the volatile matter content of the coal sample is 34.79%, which indicates that the volatile matter content of the coal sample is sufficient to cause the coal dust to explode under certain conditions. According to the ultimate analysis result of the coal sample, it can be concluded that the main components of the coal sample are carbon and oxygen, which are also the main components involved in the combustion and explosion of coal dust cloud.

**Table 1:** Main components of coking coal dust sample.

Proximate analysis (%)				Ultimate analysis (%)			
*M*	*A*	*V*	*FC*	C	H	O	N
8.61	9.03	34.79	47.57	60.39	4.18	31.75	3.68

*M* moisture, *A* ash, *V* volatile, *FC* fixed carbon.

**Figure 2:**
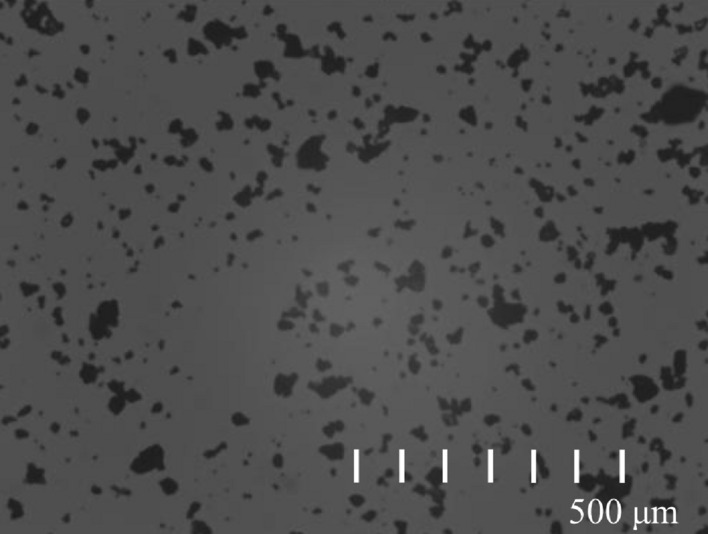
Coking coal dust particles.

**Figure 3:**
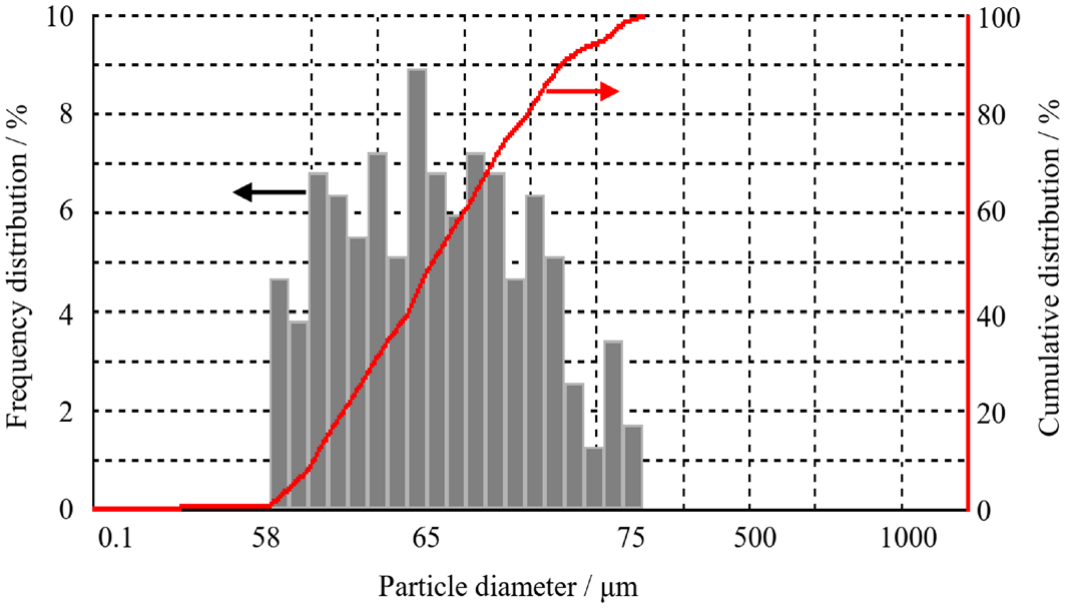
Diameter distribution of coking coal dust particle.

In addition, coal samples were also analyzed for particle size. As shown in Fig. 2, which shows the particle distribution image of the coal sample, it can be seen that the particles are relatively uniform, and there is no mutual adhesion of the particles. In Fig. 3, the particle size distribution obtained according to the particle size statistics is shown, statistical results show that the particle size of coal dust samples is greater than 58 μm and less than 75 μm, and coal dust samples within this particle size range meet the requirements of explosion experiments.

#### Inert dust samples

In the aspect of coal dust explosion suppression, the commonly used inert dusts mainly include CaCO_3_ and SiO_2_, and there are many studies on the explosion suppression effects of these two types of inert dusts. In this paper, authors consider that NaCl, KCl, and NH_4_H_2_PO_4_ in industry also have good explosion suppression effects. However, there have not been many reports on their explosion suppression effect. Therefore, NaCl, KCl, and NH_4_H_2_PO_4_ are selected as inert dust samples in this paper. In order to study the suppression effect of these three types of inert dust on coal dust explosion, the inert dust is mixed into the coal dust sample before the experiment, and then the test analysis experiment is carried out.

**Figure 4:**
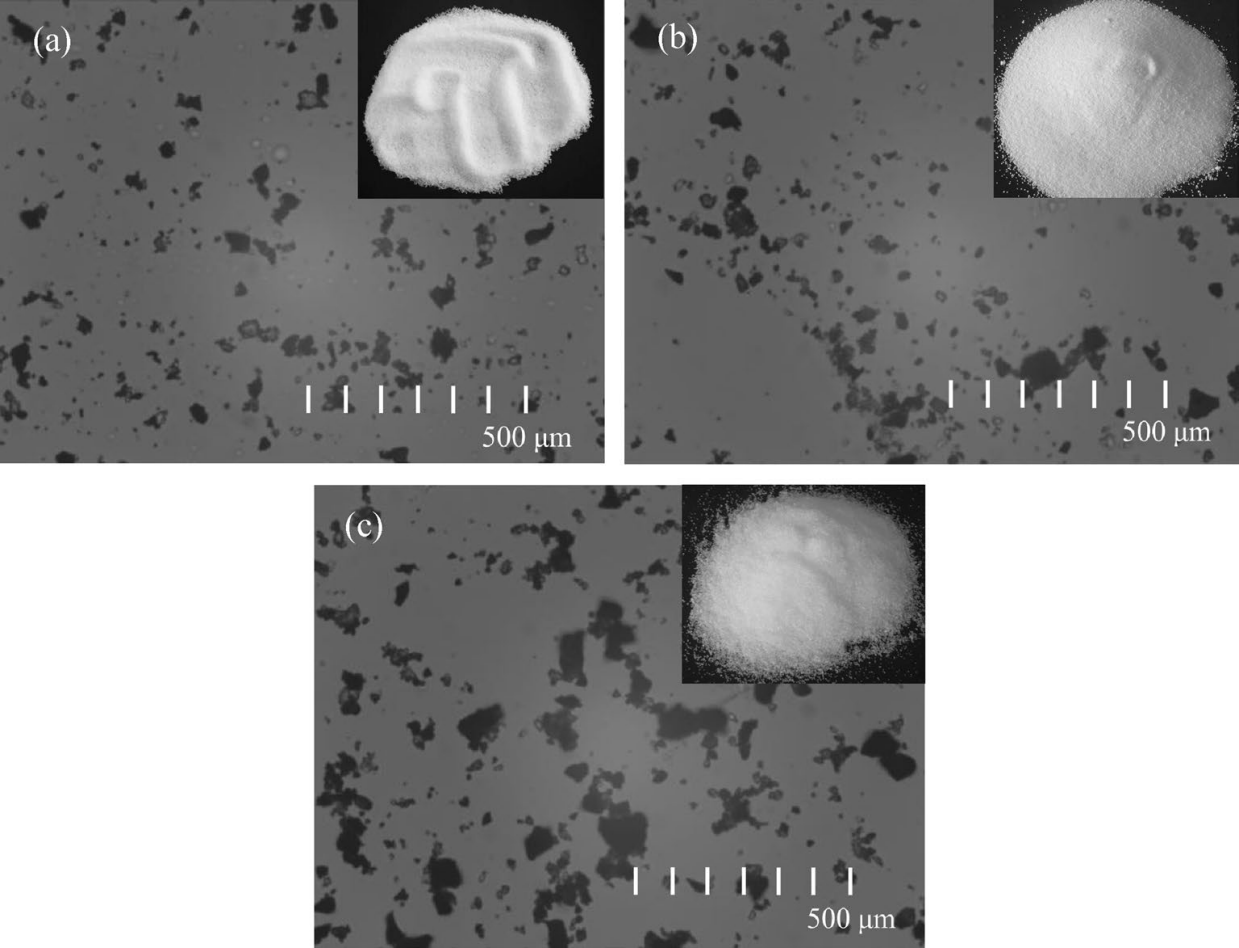
Inert dust samples. (**a**) NaCl; (**b**) KCl; (**c**) NH_4_H_2_PO_4_.

As shown in Fig. 4, it shows three types of inert dust, it can be seen that they are all white solid particles. Both NaCl and KCl have melting points greater than 1000 K, and hardly participate in the explosion reaction during the explosion process, but suppress the explosion by absorbing heat and isolating oxygen. The difference is that the melting point of NH_4_H_2_PO_4_ is less than 500 K, it can easily participate in the reaction of combustion and explosion, but when NH_4_H_2_PO_4_ is decomposed, on the one hand, it will absorb a lot of heat, and on the other hand, it will generate several explosive suppression products, Therefore, NH_4_H_2_PO_4_ is usually also used as the main component of industrial fire extinguishing agents.

## Results and discussion

### Coking coal dust explosion pressure and flame

The coal dust explosion pressure and flame experimental apparatus are used to test the coking coal dust explosion pressure and flame data, the experimental results are shown in Table 2. When testing the explosion pressure and flame, the particle size of coal dust is 58 ~ 75 μm, and the mass concentration of the coal dust cloud is 400 g/m^3^. It can be seen from Table 2 that the maximum pressure of coal dust explosion is 0.59 MPa, and the maximum length of flame propagation is 0.61 m, which indicates that under the current explosion conditions, coking coal dust exploded with a certain intensity.

**Table 2:** Coking coal dust explosion pressure and flame.

Type of coal sample	*r* (μm)	*c* (g/m^3^)	Explosion pressure and flame	
			*P*_max_ (MPa)	*l*_max_ (m)
Coking coal dust	58 ~ 75	400	0.59	0.61

*r* particle size of coal dust, *c* coal dust cloud mass concentration, *P*_max_ maximum pressure, *l*_max_ maximum flame length.

### Influence of particle size and coal dust cloud mass concentration on explosion intensity

Since there are many factors affecting the explosion pressure and flame of coal dust, in order to study the effect of particle size and coal dust cloud mass concentration on explosion intensity, different coal dust particle size and coal dust cloud mass concentration are selected for further experiments. The experimental results are shown in Table 3. It can be found that when the mass concentration of the coal dust cloud remains unchanged, the changes of the maximum pressure and the maximum flame length of the coal dust explosion are regular. When the coal dust particle size is 38 ~ 48 μm, the explosion intensity is at its maximum. At the same time, when the coal dust particle size remains unchanged, the influence of coal dust cloud mass concentration on the explosion pressure and flame is also regular. The analysis shows that when the coal dust cloud mass concentration is 400 g/m^3^, the explosion intensity is the largest. Based on the above analysis, it can be concluded that when the coal dust particle size is 38 ~ 48 μm, and the coal dust cloud mass concentration is 400 g/m^3^, the explosion pressure and the flame intensity reach the maximum value, and the maximum pressure and the maximum flame length are 0.68 MPa and 0.74 m.

**Table 3:** Influence of particle size and coal dust cloud mass concentration on explosion pressure and flame.

*r* (μm)	*c* (g/m^3^)	Explosion pressure and flame		*r* (μm)	*c* (g/m^3^)	Explosion pressure and flame	
		*P*_max_ (MPa)	*l*_max_ (m)			*P*_max_ (MPa)	*l*_max_ (m)
58 ~ 75	300	0.51	0.52	25 ~ 38	400	0.62	0.66
48 ~ 58	300	0.54	0.57	0 ~ 25	400	0.55	0.59
38 ~ 48	300	0.60	0.65	58 ~ 75	450	0.53	0.56
25 ~ 38	300	0.54	0.57	48 ~ 58	450	0.57	0.60
0 ~ 25	300	0.46	0.50	38 ~ 48	450	0.62	0.69
58 ~ 75	350	0.56	0.58	25 ~ 38	450	0.56	0.61
48 ~ 58	350	0.60	0.62	0 ~ 25	450	0.49	0.54
38 ~ 48	350	0.65	0.70	58 ~ 75	500	0.46	0.50
25 ~ 38	350	0.59	0.63	48 ~ 58	500	0.50	0.55
0 ~ 25	350	0.52	0.56	38 ~ 48	500	0.55	0.64
58 ~ 75	400	0.59	0.61	25 ~ 38	500	0.49	0.56
48 ~ 58	400	0.63	0.65	0 ~ 25	500	0.43	0.50
38 ~ 48	400	0.68	0.74	–	–	–	–

*r* particle size of coal dust, *c* coal dust cloud mass concentration, *P*_max_ maximum pressure, *l*_max_ maximum flame length.

**Figure 5:**
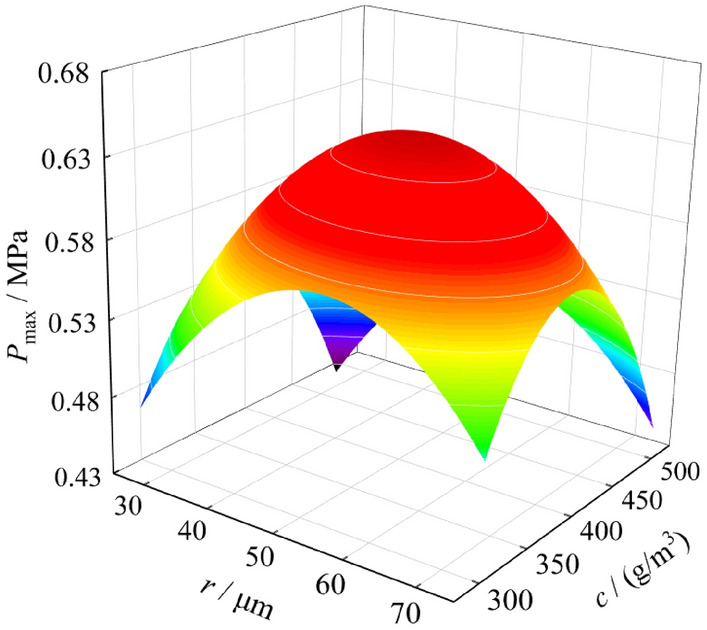
Fitting surface of explosion pressure.

**Figure 6:**
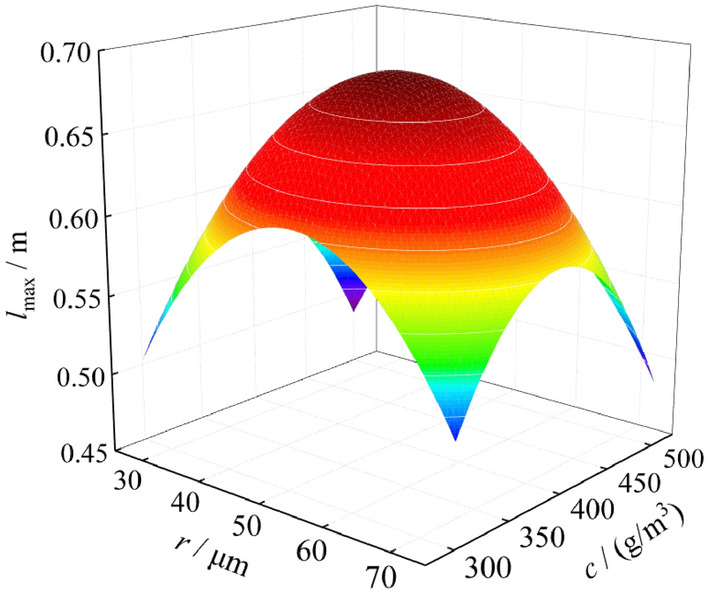
Fitting surface of explosion flame.

In order to more intuitively analyze the influence of particle size and coal dust cloud mass concentration on explosion pressure and flame, three-dimensional fitting surfaces of explosion pressure and flame are drawn, as shown in Figs. 5 and 6, respectively. There are two independent variables for the fitted surface, namely particle size and coal dust cloud mass concentration, and the dependent variables are the maximum pressure and the maximum flame length, respectively. It can be found that the three-dimensional surfaces of the maximum pressure and the maximum flame length have a maximum value point, indicating that the particle size and the mass concentration of coal dust cloud have a great influence on the explosion pressure and flame, and there is an optimal particle size and an optimal coal dust cloud mass concentration. Under this optimal condition, the maximum pressure and the maximum flame length of coal dust explosion both reach the maximum value on the three-dimensional surface.

### Suppression effect of inert dust on coking coal dust explosion pressure and flame

The research results in “Influence of particle size and coal dust cloud mass concentration on explosion intensity” section show that coking coal dust explosion is very dangerous, so the authors in this section will carry out research on the suppression of coking coal dust explosion by inert dust. In this part of the experiment, the particle size of coking coal dust is 38 ~ 48 μm, and the mass concentration of coal dust cloud is 400 g/m^3^. Because the explosion intensity is the largest under this condition, the effect of research on explosion suppression is more obvious. The three inert dusts selected are NaCl, KCl, and NH_4_H_2_PO_4_, respectively. The particle size of inert dust is the same as that of coking coal dust, which is also 38 ~ 48 μm. The main reason for selecting these three types of inert dusts is that they are all inert dusts capable of suppressing explosions. At the same time, there have not been a large number of reports on the research results of their explosion suppression effects on coking coal dust. The experimental results of explosion suppression are shown in Table 4. It can be found that among the three inert dusts, NH_4_H_2_PO_4_ has the best explosion suppression effect. When the mass percentage of NH_4_H_2_PO_4_ mixed into the coking coal dust is 70%, the explosion will no longer occur.

**Table 4:** Suppression effect of inert dust on coking coal dust explosion pressure and flame.

*p* (%)	NaCl		KCl		NH_4_H_2_PO_4_	
	*P*_max_ (MPa)	*l*_max_ (m)	*P*_max_ (MPa)	*l*_max_ (m)	*P*_max_ (MPa)	*l*_max_ (m)
0	0.68	0.74	0.68	0.74	0.68	0.74
10	0.64	0.69	0.62	0.66	0.60	0.62
20	0.56	0.65	0.54	0.60	0.51	0.56
30	0.48	0.61	0.45	0.56	0.43	0.47
40	0.42	0.56	0.39	0.50	0.36	0.40
50	0.37	0.50	0.34	0.43	0.30	0.32
60	0.33	0.44	0.29	0.37	0.21	0.24
70	0.25	0.37	0.23	0.31	0	0

*p* mass percentage of inert dust mixed into coking coal dust.

**Figure 7:**
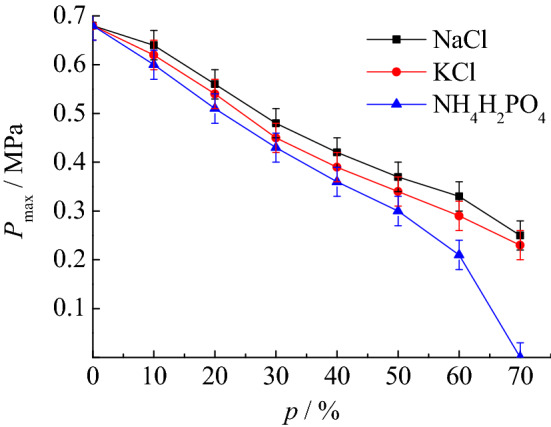
Suppression effect of inert dust on *P*_max_.

**Figure 8:**
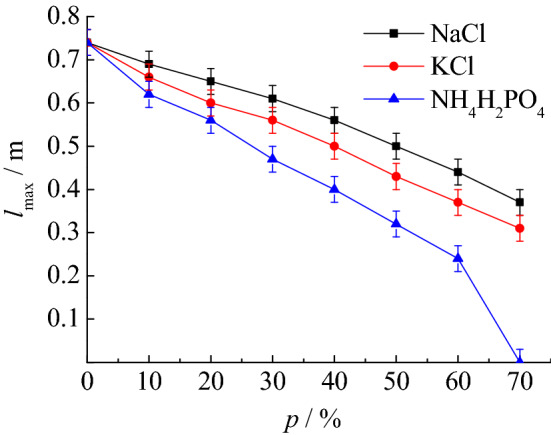
Suppression effect of inert dust on *l*_max_.

According to the experimental data, the explosion suppression curves of three types of inert dusts were drawn. As shown in Figs. 7,8, each experiment is carried out 10 times, and the average value of the ten experiments is obtained as the final experimental result, and error bars are also drawn on the Figs. 7,8. It can be seen that as the mass percentage of the inert dust mixed into the coking coal increases, the maximum explosion pressure and the maximum flame length decrease continuously. According to the explosion suppression curves, it can be found that the explosion suppression effect of NaCl is not as good as that of KCl, and the explosion suppression effect of KCl is not as good as that of NH_4_H_2_PO_4_. When the mass percentage of NH_4_H_2_PO_4_ mixed into coking coal dust increases from 0 to 60%, the maximum explosion pressure decreases by 0.47 MPa, and the maximum flame length of the explosion decreases by 0.50 m, which indicates that the suppression effect of NH_4_H_2_PO_4_ on coking coal dust explosion is very obvious. Next, the authors will discuss the influence of particle size of NH_4_H_2_PO_4_ on explosion suppression in the next section.

### Influence of particle size of NH_4_H_2_PO_4_ on suppression of coking coal dust explosion

**Figure 9:**
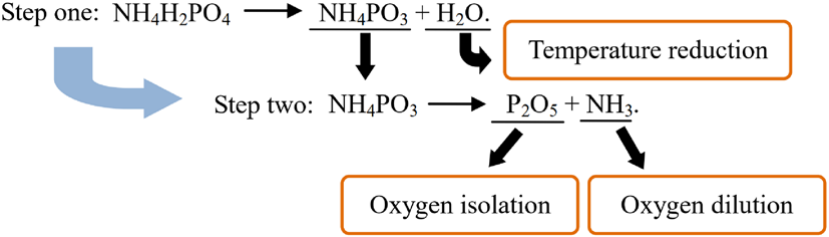
Explosion suppression process of inert dust NH_4_H_2_PO_4_.

In the experiment in this section, in order to study the influence of particle size of NH_4_H_2_PO_4_ on explosion suppression, NH_4_H_2_PO_4_ with different particle sizes was selected as inert dust. The particle size of coking coal dust is 38 ~ 48 μm, and the mass concentration of coal dust cloud is 400 g/m^3^. The experimental results of explosion suppression are shown in Table 5. It can be seen that the smaller the particle size of NH_4_H_2_PO_4_, the greater the suppression effect of coking coal dust explosion pressure and flame. When the particle size of NH_4_H_2_PO_4_ is 25 ~ 38 μm, and the mass percentage of inert dust mixed into the coking coal dust is 60%, the explosion will no longer occur. When the particle size of NH_4_H_2_PO_4_ is 0 ~ 25 μm, and the mass percentage of inert dust mixed into coking coal dust is 50%, the explosion will no longer occur. According to the principle of reaction kinetics, it can be obtained that NH_4_H_2_PO_4_ will undergo the following chemical reactions during the explosion. As shown in Fig. 9, in the process of suppressing the explosion, NH_4_H_2_PO_4_ will first generate H_2_O and NH_4_PO_3_, and H_2_O will absorb a lot of heat, which is conducive to suppressing the explosion reaction. NH_4_PO_3_ will further generate P_2_O_5_ and NH_3_, which can isolate oxygen and dilute the concentration of oxygen respectively, so that the explosion intensity of coking coal dust is greatly reduced.

**Table 5:** Influence of particle size of NH_4_H_2_PO_4_ on coking coal dust explosion pressure and flame.

*p* (%)	0 ~ 25 μm		25 ~ 38 μm		38 ~ 48 μm		48 ~ 58 μm		58 ~ 75 μm	
	*P*_max_ (MPa)	*l*_max_ (m)	*P*_max_ (MPa)	*l*_max_ (m)	*P*_max_ (MPa)	*l*_max_ (m)	*P*_max_ (MPa)	*l*_max_ (m)	*P*_max_ (MPa)	*l*_max_ (m)
0	0.68	0.74	0.68	0.74	0.68	0.74	0.68	0.74	0.68	0.74
10	0.56	0.57	0.57	0.60	0.60	0.62	0.62	0.65	0.64	0.67
20	0.45	0.50	0.49	0.53	0.51	0.56	0.54	0.58	0.55	0.60
30	0.36	0.41	0.40	0.44	0.43	0.47	0.46	0.49	0.48	0.51
40	0.29	0.31	0.33	0.36	0.36	0.40	0.38	0.43	0.39	0.45
50	0	0	0.26	0.30	0.30	0.32	0.33	0.34	0.35	0.37
60	0	0	0	0	0.21	0.24	0.24	0.26	0.26	0.28
70	0	0	0	0	0	0	0	0	0	0

*p* mass percentage of inert dust mixed into coal dust.

**Figure 10:**
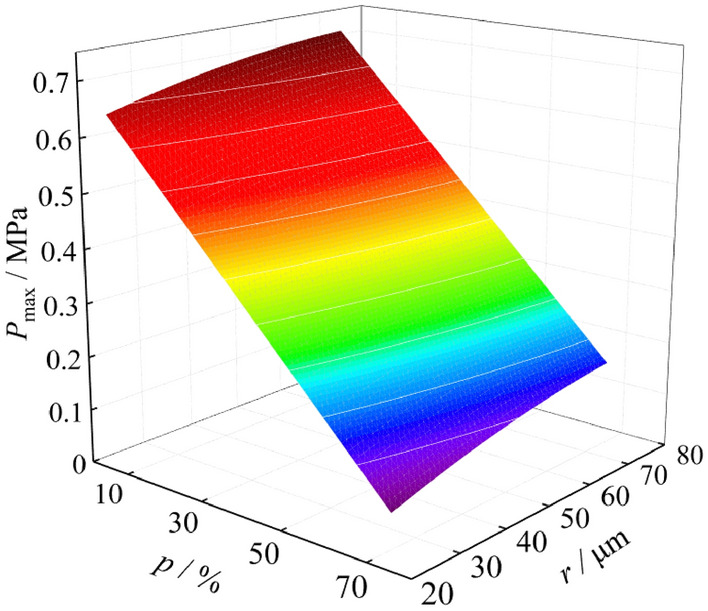
Suppression effect of NH_4_H_2_PO_4_ on *P*_max_.

**Figure 11:**
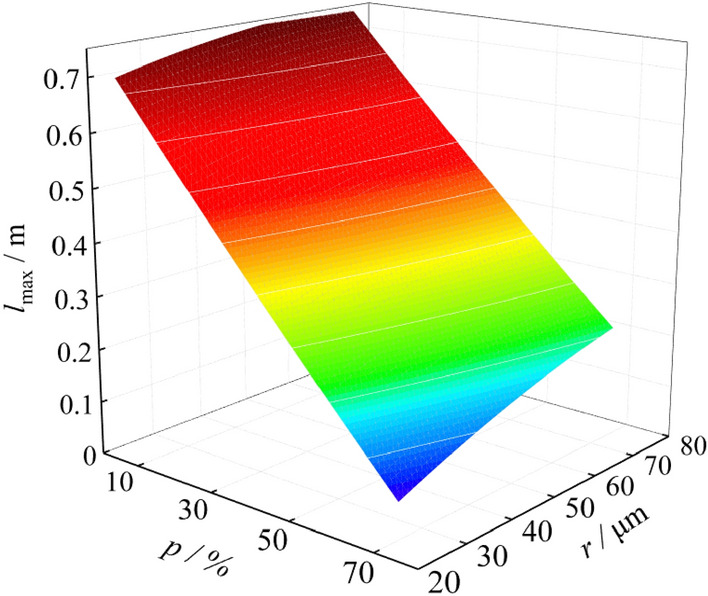
Suppression effect of NH_4_H_2_PO_4_ on *l*_max_.

According to the experimental data, the three-dimensional fitting surfaces of explosion suppression of NH_4_H_2_PO_4_ are drawn. As shown in Figs. 10,11, there are two independent variables for fitting the surfaces, which are the mass percentage of NH_4_H_2_PO_4_ mixed into the coking coal dust and the particle size of NH_4_H_2_PO_4_. The dependent variables of the fitted surfaces are the maximum pressure of the explosion and the maximum flame length, respectively. It can be seen that as the mass percentage of NH_4_H_2_PO_4_ mixed into the coking coal dust continues to increase, and as the particle size of NH_4_H_2_PO_4_ continues to decrease, the maximum explosion pressure and the maximum flame length continue to decrease, which shows that the intensity of the explosion is effectively suppressed. When the particle size of NH_4_H_2_PO_4_ is 58 ~ 75 μm, and the mass percentage of NH_4_H_2_PO_4_ mixed into coking coal dust is 40%, the maximum explosion pressure and maximum flame length are reduced by 0.29 MPa and 0.29 m, respectively. When the particle size of NH_4_H_2_PO_4_ is 0 ~ 25 μm, and the mass percentage of NH_4_H_2_PO_4_ mixed into coking coal dust is 40%, the maximum explosion pressure and maximum flame length are reduced by 0.39 MPa and 0.43 m, respectively. This shows that the reduction of the particle size of the NH_4_H_2_PO_4_ plays an important role in suppressing the explosion intensity.

## Conclusions

In this paper, the influencing factors of coking coal dust explosion intensity are discussed, and the suppression effect of inert dust on coking coal dust explosion pressure and flame is studied. The conclusions are as follows.

By studying the influencing factors of coking coal dust explosion pressure and flame, it is found that the particle size and the mass concentration of coal dust have a great influence on the explosion pressure and flame. When the coal dust particle size is 38 ~ 48 μm, and the coal dust cloud mass concentration is 400 g/m^3^, the explosion intensity is the maximum, and the maximum pressure and the maximum flame length are 0.68 MPa and 0.74 m.

By analyzing the suppression effects of NaCl, KCl, and NH_4_H_2_PO_4_ on coking coal dust explosion, it is found that the explosion suppression effect of NaCl is not as good as that of KCl, and NH_4_H_2_PO_4_ has the best explosion suppression effect. When the mass percentage of NH_4_H_2_PO_4_ mixed into coking coal dust increases to 60%, the maximum explosion pressure decreases by 0.47 MPa, and the maximum flame length decreases by 0.50 m.

By discussing the influence of particle size of NH_4_H_2_PO_4_ on the explosion suppression, it is found that when the particle size of NH_4_H_2_PO_4_ is 0 ~ 25 μm, and the mass percentage of NH_4_H_2_PO_4_ mixed into coking coal dust is 50%, the explosion will no longer occur. As the mass percentage of NH_4_H_2_PO_4_ mixed into coking coal dust increases, and as the particle size of NH_4_H_2_PO_4_ decreases, the maximum explosion pressure and the maximum flame length continue to decrease, which shows that the intensity of the explosion is effectively suppressed.

## Data Availability

All data generated or analysed in this study are included in this published article.
